# Accelerated ageing and renal dysfunction links lower socioeconomic status and dietary phosphate intake

**DOI:** 10.18632/aging.100948

**Published:** 2016-04-26

**Authors:** Ruth McClelland, Kelly Christensen, Suhaib Mohammed, Dagmara McGuinness, Josephine Cooney, Andisheh Bakshi, Evangelia Demou, Ewan MacDonald, Muriel Caslake, Peter Stenvinkel, Paul G. Shiels

**Affiliations:** ^1^ Institute of Cancer Sciences, MVLS, University of Glasgow, Glasgow, UK; ^2^ School of Medicine, MVLS, University of Glasgow, Glasgow, UK; ^3^ Institute of Health and Wellbeing, MVLS, University of Glasgow, Glasgow, UK; ^4^ Division of Renal Medicine M99, Department of Clinical Science, Intervention and Technology, Karolinska Institutet, Stockholm, Sweden

**Keywords:** phosphate, diet, poverty, ageing, CKD, pSoBid

## Abstract

**Background:**

We have sought to explore the impact of dietary Pi intake on human age related health in the pSoBid cohort (n=666) to explain the disparity between health and deprivation status in this cohort. As hyperphosphataemia is a driver of accelerated ageing in rodent models of progeria we tested whether variation in Pi levels in man associate with measures of biological ageing and health.

**Results:**

We observed significant relationships between serum Pi levels and markers of biological age (telomere length (p=0.040) and DNA methylation content (p=0.028), gender and chronological age (p=0.032). When analyses were adjusted for socio-economic status and nutritional factors, associations were observed between accelerated biological ageing (telomere length, genomic methylation content) and dietary derived Pi levels among the most deprived males, directly related to the frequency of red meat consumption.

**Conclusions:**

Accelerated ageing is associated with high serum Pi levels and frequency of red meat consumption. Our data provide evidence for a mechanistic link between high intake of Pi and age-related morbidities tied to socio-economic status.

## INTRODUCTION

In the Scottish city of Glasgow, there is a large difference in life expectancy between affluent and deprived communities. This remarkable difference is one of the largest reported in the developed world, despite common risk factors for age-associated morbidities [1–4], despite the most and least deprived living within a 15 km geographical radius. This exceptional gradient of socio-economic difference is reflected in the associated variation in mortality and morbidity in this city.

The reasons for this remain unclear, but these have been investigated as a part of the psychological, social, and biological determinants of ill health (pSoBid) study cohort [1] the characteristics of which have been described in depth elsewhere [2].

In the pSoBid cohort, we have demonstrated previously that lower socio-economic status is associated with the expression level of the pro-inflammatory cytokine Interleukin 6 (IL-6), a marker of cardiovascular disease and with features of insulin resistance, such as elevated adipokine levels [3]. Additionally, we have demonstrated an association between accelerated ageing amongst the most deprived in association with poor diet, telomere shortening and genomic DNA hypomethylation [4, 5].

A common direct link between ageing and nutrition has also been demonstrated recently within mammalian genera, based around the uptake of dietary phosphate (Pi), with a strong correlation between Pi levels and longevity [6]. This is supported by data from Ohnishi et al [7, 8] who have demonstrated that Pi toxicity accelerates mammalian ageing. Recent evidence has shown that inorganic Pi shortens life span in klotho-deficient mice via activation of the AKT/mammalian target of rapamycin complex 1 (mTORC1) [9].

Phosphate is naturally present in basic foodstuffs, including meats, fish, eggs, dairy products and vegetables. Intestinal absorption of naturally occurring Pi is minimally regulated, as absorption is efficient, hence high Pi supplementation results in markedly elevated levels of serum phosphate. In the human body, Pi levels are regulated via vitamin D, parathyroid hormone (PTH) and FGF-23/klotho. High Pi levels, as a consequence of dietary intake, have been linked to higher all-cause and cardiovascular mortality risk in the NHANES III study [10] and to premature vascular ageing in the general population [11–15].

Extracellular Pi exerts cytotoxic effects by forming insoluble nanoparticles, termed calciprotein particles. Calciprotein particles are highly bioactive ligands that can induce various cellular responses, including the osteogenic transformation of vascular smooth muscle cells and the cell death of vascular endothelial and renal tubular epithelial cells [10]. Indeed, high Pi levels are a common feature of various metabolic diseases [16–18] including the rare Hutchinson-Gilford's Progeria syndrome [19].

Notably, the effects of calciprotein particles can be mitigated via the action of Fetuin A, a proven a mediator of biological ageing in a number of pathologies, including end stage renal disease and cancer [20, 21], where telomere length, a marker of biological age in circulating blood cells, is dependent on circulating Fetuin A levels.

Collectively, these observations in humans and other mammals are consistent with the hypothesis that increased Pi intake drives biological ageing processes [16, 20–24] and suggest that Pi intake may contribute to human health span at a fundamental level.

This is particularly intriguing in the context of Glasgow, as an association between serum Pi and SES has also been described previously [23], raising the possibility that the accelerated ageing observed among Glasgow's most deprived is contributed to by increased intake of dietary Pi.

We explored if variation in serum Pi levels were associated with the socioeconomic differences, accelerated ageing and epigenetic status among the participants in the pSoBid cohort. In turn, we have evaluated whether there were any associations with intake of specific dietary factors, including processed foods and their frequency of consumption.

## RESULTS

Subject characteristics relative to socioeconomic status (SES) and pertinent biochemical, biophysical lifestyle and nutritional parameters are listed in Table 1.

**Table 1:** Median (IQR) blood phosphate level (mmol/L) with subgroups of gender, socio-economic and lifestyle factors overall and by age groups

				Age group (Years)		
	Subgroup	n	All	n=193 35-44	n=193 45-54	n=215 55-64
		604	0.99 (0.86-1.13)	0.98 (0.85-1.08)	0.97 (0.85-1.13)	1.02 (0.90-1.16)
**Gender**	Female (F)	308 (51%)	1.02 (0.87-1.14)	1.04 (0.89-1.12)	1.03 (0.90-1.18)	0.98 (0.85-1.14)
	Male (M)	293 (49%)	0.97 (0.85-1.08)	0.98 (0.88-1.12)	0.96 (0.84-1.09)	0.96 (0.84-1.06)
**Deprivation**	LD (least deprived)	307 (51%)	0.97 (0.86-1.10)	0.99 (0.88-1.14)	0.97 (0.84-1.22)	0.96 (0.85-1.06)
	MD (most deprived)	294 (49%)	1.01 (0.87-1.14)	0.99 (0.86-1.18)	1.02 (0.91-1.15)	1.00 (0.85-1.15)
**Employed**	No	188 (31%)	1.01 (0.85-1.15)	1.01 (0.89-1.14)	1.01 (0.84-1.12)	0.98 (0.84-1.20)
	Yes	407 (67%)	0.99 (0.87-1.10)	0.96 (0.86-1.11)	0.99 (0.87-1.10)	1.00 (0.87-1.12)
**Household income**	<=£25K	276 (46%)	1.00 (0.86-1.14)	1.01 (0.88-1.13)	0.96 (0.82-1.11)	1.04 (0.91-1.16)
	>£25K	283 (47%)	0.97 (0.85-1.10)	1.00 (0.86-1.11)	0.99 (0.85-1.09)	0.96 (0.87-1.09)
**Smoking**	Current	134 (22%)	1.00 (0.87-1.13)	0.99 (0.91-1.17)	1.00 (0.87-1.07)	1.02 (0.84-1.21)
	Former	157 (26%)	1.02 (0.88-1.14)	1.01 (0.84-1.14)	0.99 (0.86-1.13)	1.04 (0.92-1.15)
	Never	279 (46%)	0.98 (0.85-1.10)	1.00 (0.87-1.12)	0.98 (0.85-1.1)	0.97 (0.86-1.09)
**Regular smoker**	No	278 (46%)	0.98 (0.98-1.10)	0.99 (087-1.1)	0.99 (0.85-1.12)	0.96 (0.86-1.09)
	Yes	323 (54%)	1.01 (0.87-1.14)	1.02 (0.95-1.13)	0.99 (0.86-1.13)	1.03 (0.86-1.14)
**High Cholesterol**	No	478 (79%)	0.99 (0.86-1.12)	0.99 (0.88-1.13)	0.99 (0.85-1.09)	0.99 (0.98-1.12)
	Yes	116 (19%)	1.00 (0.87-1.14)	0.99 (0.91-1.16)	1.05 (0.88-1.14)	0.99 (0.85-1.10)
**High BP**	No	448 (74%)	1.00 (0.87-0.10)	1.00 (0.89-1.12)	1.00 (0.88-1.11)	0.99 (0.85-1.12)
	Yes	138 (23%)	0.98 (0.84-1.14)	1.06 (0.88-1.20)	0.96 (0.82-1.09)	0.97 (0.83-1.15)
**eGFR**	Lower 50%	279 (46.2%)	1.01 (0.875-1.15)	0.975 (0.832-1.072)	1 (0.875-1.105)	1.01 (0.87-1.18)
	Upper 50%	303 (50.2%)	0.98 (0.86-1.095)	0.96 (0.857-1.08)	0.99 (0.81-1.128)	0.99 (0.887-1.08)
**Diabetes**	No	558 (93%)	0.99 (0.86-1.12)	0.97 (0.85-1.1)	0.99 (0.86-1.13)	1.00 (0.87-1.11)
	Yes	34 (6%)	1.10 (0.99-1.22)	1.03 (0.84-1.16)	1.17 (0.99-1.27)	1.08 (0.98-1.22)
**Cancer**	No	557 (92%)	0.99 (0.86-1.13)	0.96 (0.84-1.08)	1.00 (0.88-1.14)	1.00 (0.87-1.14)
	Yes	37 (6%)	1.00 (0.86-1.10)	0.84 (0.79-0.99)	0.96 (0.90-1.10)	1.08 (1.00-1.20)
**Excessive alcohol (>14 (F), >21 (M))**	No	494 (82%)	1.00 (0.86-1.13)	0.98 (0.85-1.12)	1.00 (0.85-1.12)	1.00 (0.85-1.12)
	Yes	107 (18%)	0.98 (0.89-1.08)	1.00 (0.92-1.15)	0.96 (0.88-1.03)	1.00 (0.86-1.14)
**Obese (BMI>30 kg/m^2^)**	No	428 (71%)	0.99 (0.85-1.11)	0.97 (0.84-1.12)	0.99 (0.85-1.08)	1.00 (0.87-1.12)
	Yes	175 (29%)	0.99 (0.89-1.13)	0.97 (0.86-1.13)	1.05 (0.92-1.15)	0.97 (0.86-1.13)
**Diet score**	Lower 50%	300 (50%)	0.99 (0.86-1.12)	1.00 (0.86-1.12)	0.99(.090-1.09)	0.98 (0.85-1.14)
	Upper 50%	303 (5%)	1.00 (0.87-1.13)	1.02 (0.9-1.15)	1.01 (0.87-1.13)	0.96 (0.85-1.10)
**Red meat**	G1.pD-G1.pW	355 (59%)	1 (0.9-1.14)	1 (0.91-1.11)	1 (0.88-1.14)	1 (0.87-1.12)
	L1.pW - L1.pM	137 (23%)	0.98 (0.81-1.09)	0.94 (0.8-1.06)	0.97 (0.8-1.06)	1.03 (0.9-1.18)
	G1.pM	65 (11%)	0.95 (0.83-1.13)	0.87 (0.78-1.02)	0.94 (0.79-1.06)	1.14 (0.92-1.23)
**Meat Product**	G1.pD-G1.pW	54 (9%)	1 (0.86-1.19)	0.83 (0.8-0.94)	1 (0.89-1.22)	0.98 (0.8-1.12)
	L1.pW - L1.pM	167 (28%)	1.02 (0.89-1.14)	1.02 (0.9-1.08)	0.99 (0.86-1.11)	1.07 (0.9-1.2)
	G1.pM	145 (24%)	0.99 (0.87-1.1)	1 (0.87-1.09)	0.98 (0.86-1.09)	1 (0.91-1.1)
**Cheese**	G1.pD-G1.pW	343 (57%)	0.99 (0.86-1.12)	0.96 (0.85-1.07)	0.99 (0.84-1.12)	1.04 (0.9-1.16)
	L1.pW - L1.pM	114 (19%)	0.98 (0.88-1.09)	0.97 (0.86-1.08)	1.02 (0.88-1.12)	0.97 (0.84-1.01)
	G1.pM	68 (11%)	0.97 (0.82-1.18)	0.92 (0.81-1.02)	0.94 (0.82-1.13)	1.02 (0.78-1.18)
**Soft Fizzy Drinks**	G1.pD-G1.pW	241 (40%)	0.99 (0.86-1.13)	0.99 (0.83-1.11)	0.98 (0.85-1.13)	1 (0.88-1.13)
	L1.pW - L1.pM	88 (15%)	1.02 (0.89-1.13)	0.96 (0.88-1.05)	1.02 (0.9-1.12)	1.02 (0.83-1.16)
	G1.pM	62 (10%)	0.96 (0.85-1.05)	0.92 (0.85-0.96)	0.96 (0.8-1.05)	0.97 (0.87-1.05)

LD-least deprived, MD-most deprived, eGFR-estimated glomerular filtration rate, BP-blood pressure, food intake frequency: G1.pD-G1.pW -greater than once per day –greater than once per week; L1.pW - L1.pM- less than 1 per week- no greater than 1 month; G1.pM –greater than 1 per month. Alcohol consumption is defined relative to recommended units per week.

**Determination of associations between serum phosphate levels, SES factors, biomarkers of ageing and biochemical parameters associated with chronic inflammation and metabolic syndrome**.

In unadjusted analyses Pi levels showed significant associations with age (r= 0.09, 95% CI 0.008 to 0.167; p=0.032), log relative telomere length (95%CI −0.224 to −0.02; p=0.040), global DNA methylation content (95%CI −0.284 to −0.03; p=0.028), IL-6 (95%CI 0.017 to 0.179; p=0.043), eGFR (95%CI −0.186 to −0.026; p=0.025), adiponectin (95%CI 0.016 to 0.176; p=0.043) and D-dimer (95%CI 0.027 to 0.188; p=0.025)(Table 2), in keeping with previous data showing a relationship between features of biological ageing and deprivation in this cohort.

**Table 2:** Analysis of phosphate levels versus telomere length, DNA methylation content, biochemical parameters and income, with data adjusted for age, gender and deprivation status

Adjustment Covariates/*stratified variable*	Relative Telomere length	Global DNA methylation	Adiponectin	Cholesterol	Interleukin 6	eGFR (comb.ckdepi)	D-dimer	Vitamin D
**None**	(n=360) r=−0.124 (−0.224 to −0.02) p=0.041*	(n=229) r=−0.16 (−0.284 to −0.031) p=0.028*	(n=586) r=0.097 (0.016 to 0.176) p=0.043*	(n=589) r=0.081 (0 to 0.16) p=0.095	(n=574) r=0.099 (0.017 to 0.179) p=0.043*	(n=582) r=−0.107 (−0.186 to −0.026) p=0.025*	(n=583) r=0.108 (0.027 to 0.188) p=0.025*	(n=570) r=−0.064 (−0.145 to 0.018) p=0.240
**Age**	(n=360) r=−0.149 (−0.248 to −0.046) p=0.040*	(n=229) r=−0.233 (−0.352 to −0.106) p=0.012*	(n=586) r=0.126 (0.045 to 0.205) p=0.027*	(n=589) r=0.121 (0.041 to 0.2) p=0.037*	(n=574) r=0.118 (0.037 to 0.198) p=0.043*	(n=582) r=−0.117 (−0.196 to −0.036) p=0.040*	(n=583) r=0.126 (0.045 to 0.205) p=0.025*	(n=570) r=−0.124 (−0.204 to −0.042) p=0.034*
**Gender**	(n=359) r=−0.162 (−0.261 to −0.059) p=0.036*	(n=228) r=−0.174 (−0.297 to −0.045) p=0.054	(n=584) r=0.132 (0.052 to 0.211) p=0.025*	(n=587) r=0.141 (0.061 to 0.22) p=0.016*	(n=572) r=0.168 (0.087 to 0.247) p=0.003**	(n=580) r=−0.147 (−0.226 to −0.067) p=0.015*	(n=581) r=0.148 (0.067 to 0.226) p=0.011*	(n=568) r=−0.137 (−0.217 to −0.055) p=0.016*
*Male*	(n=191) r=−0.18 (−0.314 to −0.039) p=0.036*	(n=113) r=−0.029 (−0.212 to 0.157) p=0.764	(n=290) r=0.037 (−0.079 to 0.151) p=0.591	(n=290) r=−0.013 (−0.128 to 0.102) p=0.826	(n=284) r=0.115 (−0.002 to 0.228) p=0.101	(n=287) r=0.008 (−0.108 to 0.124) p=0.944	(n=287) r=0.093 (−0.023 to 0.207) p=0.164	(n=280) r=−0.183 (−0.294 to −0.067) p=0.010*
*Female*	(n=168) r=−0.08 (−0.229 to 0.072) p=0.368	(n=115) r=−0.282 (−0.442 to −0.104) p=0.012*	(n=294) r=0.063 (−0.051 to 0.177) p=0.473	(n=297) r=0.155 (0.042 to 0.265) p=0.027*	(n=288) r=0.098 (−0.018 to 0.211) p=0.149	(n=293) r=−0.154 (−0.264 to −0.04) p=0.025*	(n=294) r=0.084 (−0.031 to 0.197) p=0.197	(n=288) r=0.03 (−0.086 to 0.145) p=0.691
**Deprivation**	(n=359) r=−0.156 (−0.255 to −0.053) p=0.036*	(n=228) r=−0.205 (−0.326 to −0.077) p=0.019*	(n=584) r=0.115 (0.035 to 0.195) p=0.043*	(n=587) r=0.108 (0.027 to 0.187) p=0.069	(n=572) r=0.103 (0.021 to 0.184) p=0.102	(n=580) r=−0.115 (−0.195 to −0.034) p=0.034*	(n=581) r=0.115 (0.034 to 0.194) p=0.041*	(n=568) r=−0.075 (−0.157 to 0.007) p=0.333
*Most Deprived*	(n=186) r=−0.138 (−0.276 to 0.006) p=0.094	(n=120) r=−0.045 (−0.223 to 0.135) p=0.661	(n=287) r=0.046 (−0.07 to 0.161) p=0.591	(n=288) r=0.075 (−0.041 to 0.189) p=0.267	(n=278) r=0.102 (−0.016 to 0.217) p=0.149	(n=286) r=−0.113 (−0.226 to 0.003) p=0.086	(n=282) r=0.051 (−0.066 to 0.167) p=0.447	(n=277) r=0.016 (−0.102 to 0.133) p=0.845
*Least Deprived*	(n=173) r=−0.099 (−0.245 to 0.051) p=0.254	(n=108) r=−0.262 (−0.43 to −0.077) p=0.017*	(n=297) r=0.164 (0.051 to 0.273) p=0.025*	(n=299) r=0.106 (−0.007 to 0.217) p=0.114	(n=294) r=0.072 (−0.043 to 0.185) p=0.260	(n=294) r=−0.089 (−0.202 to 0.025) p=0.178	(n=299) r=0.148 (0.035 to 0.257) p=0.025*	(n=291) r=−0.143 (−0.254 to −0.028) p=0.036*
*Most Deprived Male*	(n=100) r=−0.256 (−0.431 to −0.063) p=0.0362*	(n=60) r=0.119 (−0.139 to 0.362) p=0.445	(n=137) r=0.029 (−0.14 to 0.196) p=0.738	(n=137) r=−0.049 (−0.215 to 0.12) p=0.624	(n=133) r=0.111 (−0.06 to 0.276) p=0.260	(n=136) r=−0.002 (−0.17 to 0.166) p=0.982	(n=134) r=−0.03 (−0.199 to 0.14) p=0.730	(n=131) r=−0.09 (−0.258 to 0.083) p=0.399
*Least Deprived Male*	(n=91) r=−0.05 (−0.253 to 0.157) p=0.678	(n=53) r=−0.086 (−0.348 to 0.189) p=0.613	(n=153) r=0.054 (−0.105 to 0.211) p=0.592	(n=153) r=0.09 (−0.07 to 0.245) p=0.325	(n=151) r=0.042 (−0.118 to 0.201) p=0.606	(n=151) r=0.062 (−0.099 to 0.219) p=0.512	(n=153) r=0.173 (0.014 to 0.322) p=0.056	(n=149) r=−0.247 (−0.392 to −0.089) p=0.010*
*Most Deprived Female*	(n=86) r=0.003 (−0.209 to 0.215) p=0.976	(n=60) r=−0.191 (−0.424 to 0.066) p=0.203	(n=150) r=0.049 (−0.113 to 0.207) p=0.591	(n=151) r=0.193 (0.034 to 0.342) p=0.042*	(n=145) r=0.1 (−0.064 to 0.259) p=0.260	(n=150) r=−0.209 (−0.357 to −0.05) p=0.025*	(n=148) r=0.131 (−0.031 to 0.286) p=0.164	(n=146) r=0.103 (−0.06 to 0.261) p=0.333
*Least Deprived Female*	(n=82) r=−0.17 (−0.373 to 0.049) p=0.179	(n=55) r=−0.389 (−0.593 to −0.138) p=0.012*	(n=144) r=0.075 (−0.09 to 0.236) p=0.573	(n=146) r=0.109 (−0.054 to 0.267) p=0.267	(n=143) r=0.126 (−0.039 to 0.284) p=0.189	(n=143) r=−0.089 (−0.25 to 0.076) p=0.376	(n=146) r=0.044 (−0.12 to 0.205) p=0.639	(n=142) r=−0.067 (−0.23 to 0.098) p=0.517
**Age + Gender**	(n=359) r=−0.185 (−0.283 to −0.083) p=0.0362*	(n=228) r=−0.242 (−0.361 to −0.116) p=0.012*	(n=584) r=0.158 (0.078 to 0.236) p=0.020*	(n=587) r=0.169 (0.089 to 0.246) p=0.006**	(n=572) r=0.18 (0.099 to 0.258) p=0.003**	(n=580) r=−0.16 (−0.239 to −0.08) p=0.015*	(n=581) r=0.163 (0.083 to 0.242) p=0.011*	(n=568) r=−0.174 (−0.253 to −0.093) p=0.010*
**Age + Gender + Deprivation**	(n=359) r=−0.207 (−0.304 to −0.106) p=0.036*	(n=228) r=−0.275 (−0.391 to −0.151) p=0.012*	(n=584) r=0.168 (0.088 to 0.245) p=0.020*	(n=587) r=0.181 (0.101 to 0.258) p=0.006**	(n=572) r=0.181 (0.101 to 0.26) p=0.004**	(n=580) r=−0.166 (−0.244 to −0.086) p=0.016*	(n=581) r=0.169 (0.089 to 0.247) p=0.011*	(n=568) r=−0.177 (−0.256 to −0.096) p=0.010*
**Household Income**	(n=360) r=−0.159 (−0.258 to −0.056) p=0.053	(n=229) r=−0.216 (−0.336 to −0.089) p=0.028*	(n=586) r=0.142 (0.062 to 0.22) p=0.027*	(n=589) r=0.142 (0.061 to 0.22) p=0.027*	(n=574) r=0.146 (0.065 to 0.225) p=0.027*	(n=582) r=−0.151 (−0.23 to −0.071) p=0.017*	(n=583) r=0.155 (0.075 to 0.234) p=0.011*	(n=570) r=−0.121 (−0.201 to −0.039) p=0.082
*High Household income (>*£*25,000)*	(n=176) r=−0.072 (−0.217 to 0.077) p=0.391	(n=115) r=−0.167 (−0.34 to 0.016) p=0.114	(n=277) r=0.106 (−0.012 to 0.221) p=0.149	(n=279) r=0.033 (−0.085 to 0.15) p=0.623	(n=273) r=0.049 (−0.07 to 0.167) p=0.443	(n=273) r=−0.051 (−0.169 to 0.068) p=0.486	(n=277) r=0.147 (0.03 to 0.26) p=0.030*	(n=269) r=−0.067 (−0.185 to 0.053) p=0.385
*Low Household income (<*£*25,000)*	(n=172) r=−0.152 (−0.295 to −0.003) p=0.078	(n=106) r=−0.091 (−0.277 to 0.102) p=0.445	(n=268) r=0.036 (−0.084 to 0.155) p=0.591	(n=269) r=0.082 (−0.038 to 0.2) p=0.267	(n=260) r=0.147 (0.026 to 0.264) p=0.044*	(n=268) r=−0.125 (−0.241 to −0.005) p=0.069	(n=265) r=0.063 (−0.058 to 0.182) p=0.370	(n=262) r=0.001 (−0.12 to 0.122) p=0.984

Analysis adjusted for the multiple comparisons using the Benjamini-Hochberg method.

eGFR - estimated glomerular filtration rate.

Adjustment covariates (i.e., gender) included both genders in the analysis, this was further followed by sample stratification by each gender individually (only male or only female) to account for the differences observed for the covariates overall.

These parameters remained significant after adjustment for age alone, in combination with gender and/or deprivation status (Table 2) again in keeping with previous data showing a relationship between features of biological ageing and deprivation in this cohort. Other factors including smoking status, alcohol consumption, high blood pressure, incidence of cancer and diabetes were additionally analyzed in the context of aging markers and biochemical parameters (Supplementary Table 1). Negative correlations were observed between Pi levels and telomere length, global DNA methylation content and eGFR, while positive correlations were observed with age in males, adiponectin, cholesterol, IL-6 and D-dimer.

No overt associations were observed with diet or other SES measures in unadjusted analyses, nor were any observed between Pi and vitamin D in unadjusted analyses. When adjusted for age and gender, however, the relationship between Pi and vitamin D was significant (95%CI −0.253 to −0.093; p=0.010). No overt relationship was observed between vitamin D levels and deprivation status, however a significant association with Pi level was noted for the least deprived males (95%CI −0.392 to −0.089; p=0.010).

**Figure 1:**
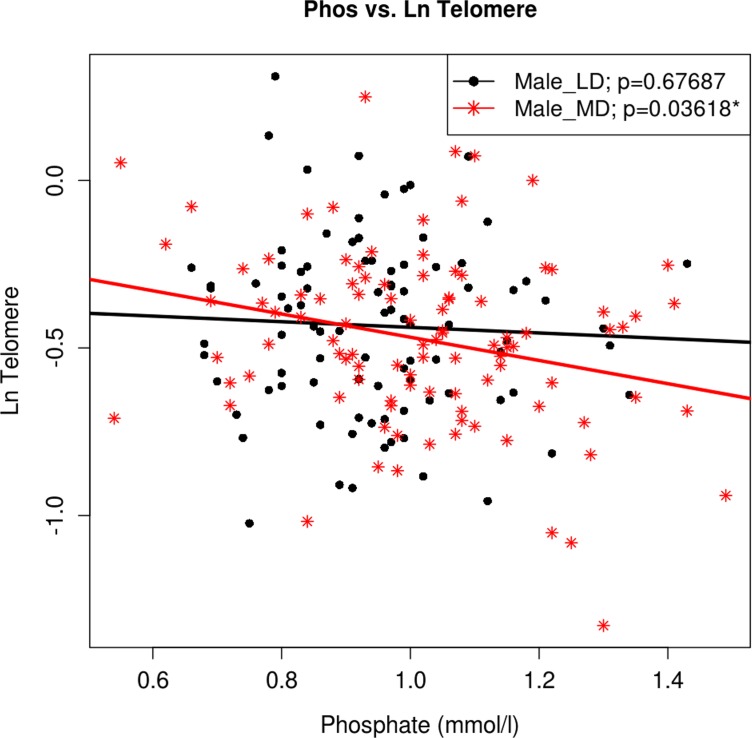
The association between Ln (Tl) and Pi in sub-samples of gender and deprivation Regression analysis of Pi levels versus telomere length adjusted for gender and deprivation status. Regression lines for males split by derivation status are colour coded and shown in the accompanying key. Male_LD - male least deprived, Male_MD - male most deprived.

The relationship between serum Pi and relative telomere length was explained by age and gender (95%CI −0.283 to −0.083; p=0.036) as expected on the basis of previous analyses [4]. Women displayed higher Pi levels than men (1.02 mmol/L vs. 0.97 mmol/L; p=0.004), though only males showed a significant association between Pi levels and chronological age, in keeping with the most deprived males having the shortest telomeres and highest serum Pi (95%CI 0.431 to −0.063; p=0.036) (Figure 1).

Similarly, differences observed in global DNA methylation content were explained by age, gender and deprivation (Table 2). Notably, the least deprived females had higher DNA methylation content (95%CI −0.593 to −0.138; p=0.012) associated with lower Pi levels, than with their most deprived counterparts and with males, again in keeping with previous observations [5]. A negative association was observed between serum Pi levels and relative telomere length and DNA methylation status in the group of regular smokers (p<0.05, Supplementary Table 1). Similarly, subjects with cancer also displayed a significant association. However, due to the small number of such individuals, this association requires further investigation and may be a type 1 statistical error.

A weak correlation was observed between Pi and adiponectin levels (Table 2). This relationship was strengthened when adjusted for age (95%CI 0.045 to 0.205; p=0.026) and gender (95%CI 0.052 to 0.211), p=0.025). The gender effect was principally explained by deprivation status (95%CI 0.035 to 0.195; p=0.043) and most prevalent among the least deprived (95%CI Additionally, the association between Pi and cholesterol was principally explained by age and gender (95%CI 0.089 to 0.246; p=0.006), with female gender being concomitant with higher cholesterol (95%CI 0.042 to 0.265; p=0.027). This relationship was not weakened when additionally adjusted for deprivation status. Indeed, in the most deprived females high cholesterol levels correlated with high Pi (95%CI 0.034 to 0.342; p=0.042).

Phosphate levels also showed an association with IL-6 levels, which was strengthened when adjusted for gender (95%CI 0.087 to 0.247; p=0.002, Table 2).

A similar circumstance was also observed for the relationship between Pi and D-dimer (p=0.025); this association was strengthened when adjusted for age, gender and deprivation (95%CI 0.089 to 0.247; p=0.011).

No association between serum Pi levels and biochemical and ageing markers were observed in relation to alcohol consumption, high blood pressure, or diabetes. In contrast ageing markers (telomeres, global DNA methylation content) were negatively correlated with the serum Pi levels, while other biochemical para-meters displayed positive correlations (Sup Table 1).

### Serum phosphate levels are affected by nutritional intake

**Figure 2:**
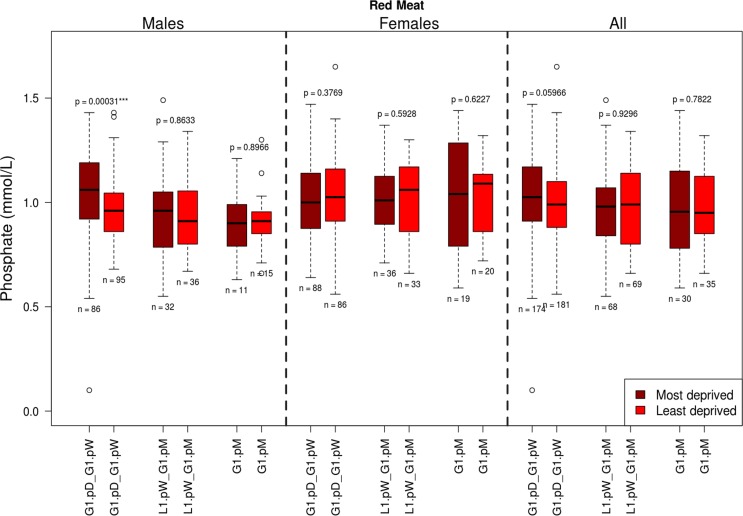
The influence of red meat consumption frequency on phosphate levels split by gender and deprivation presented as quartiles with ranges G1.pD-G1.pW =greater than once per day –greater than once per week; L1.pW - L1.pM = less than once per week – no greater than once per month; * G1.pM = greater than once per month.

Analyses of dietary factors by type and frequency of consumption (Table 1) for associations with Pi, revealed interaction between Pi levels, red meat and with fizzy drink consumption (Table 3, Figure 2).

**Table 3:** Analysis of serum phosphate levels (as a percentage increase) in relation to the frequency of foodstuffs consumption. p-adjusted p-values

Adjustment Covariates	Red Meat	Meat Product	Cheese	Fizzy Drink
**None**	(n=557) X(2)=4.096 (0.374 to 17.211) diff=0% p=0.258	(n=366) X(2)=1.223 (0.103 to 11.336) diff=0% p=0.724	(n=525) X(2)=0.201 (0.059 to 8.653) diff=0% p=0.904	(n=391) X(2)=4.808 (0.536 to 16.813) diff=0% p=0.258
**Male**	(n=275) X(2)=12.282 (3.235 to 29.711) diff=−3% p=0.008**	(n=189) X(2)=0.099 (0.06 to 7.864) diff=−3% p=0.952	(n=260) X(2)=2.622 (0.175 to 13.969) diff=−1.02% p=0.538	(n=200) X(2)=0.891 (0.076 to 9.611) diff=−2.02% p=0.855
**Female**	(n=282) X(2)=0.104 (0.054 to 8.339) diff=3% p=0.949	(n=177) X(2)=2.903 (0.254 to 14.527) diff=5% p=0.312	(n=265) X(2)=3.409 (0.225 to 17.328) diff=3.061% p=0.312	(n=191) X(2)=12.242 (4.048 to 27.127) diff=5.051% p=0.008**
**Most deprived**	(n=272) X(2)=4.666 (0.487 to 18.313) diff=1% p=0.38	(n=143) X(2)=3.319 (0.272 to 15.582) diff=4% p=0.38	(n=243) X(2)=1.883 (0.174 to 12.506) diff=1.02% p=0.52	(n=203) X(2)=0.262 (0.058 to 6.968) diff=3.03% p=0.877
**Least deprived**	(n=285) X(2)=0.683 (0.071 to 10.415) diff=−1% p=0.711	(n=223) X(2)=3.615 (0.323 to 14.888) diff=−1% p=0.328	(n=282) X(2)=1.061 (0.097 to 10.797) diff=0.51% p=0.711	(n=188) X(2)=9.012 (1.759 to 24.632) diff=−2.525% p=0.044*
**Most deprived male**	(n=129) X(2)=11.93 (3.352 to 27.448) diff=2% p=0.012*	(n=74) X(2)=0.267 (0.066 to 8.587) diff=2.5% p=0.875	(n=118) X(2)=7.036 (1.639 to 17.702) diff=1.531% p=0.06	(n=90) X(2)=2.588 (0.181 to 12.162) diff=3.03% p=0.365
**Most deprived female**	(n=143) X(2)=0.045 (0.061 to 8.675) diff=1% p=0.978	(n=69) X(2)=5.326 (0.888 to 16.014) diff=4% p=0.28	(n=125) X(2)=2.496 (0.141 to 15.814) diff=0% p=0.574	(n=113) X(2)=1.171 (0.076 to 9.169) diff=4.04% p=0.743
**Least deprived male**	(n=146) X(2)=2.5 (0.204 to 13.734) diff=−5% p=0.859	(n=115) X(2)=0.372 (0.056 to 7.985) diff=−4% p=0.859	(n=142) X(2)=0.371 (0.066 to 8.909) diff=−2.041% p=0.859	(n=110) X(2)=0.304 (0.062 to 8.459) diff=−5.556% p=0.859
**Least deprived female**	(n=139) X(2)=0.086 (0.051 to 7.585) diff=6% p=0.958	(n=108) X(2)=2.884 (0.2 to 14.332) diff=6% p=0.474	(n=140) X(2)=1.279 (0.096 to 10.848) diff=6.633% p=0.703	(n=78) X(2)=14.587 (5.49 to 28.458) diff=7.071% p=0.004**

Notably, higher Pi levels were linked to more frequent red meat consumption in males only (Kruskal-Wallis test, p=0.008, Table 3). When adjusted for deprivation status this association persisted only in the most deprived males (Kruskal-Wallis test, p=0.012, Table 3). No changes were observed for females, or least deprived males, in relation to red meat consumption.

A 5.2% increase in Pi levels was observed in the most deprived males in relation to the whole male population, with a 7.4% difference observed in Pi levels between the least and most deprived males in the pSoBid cohort.

Notably, the least deprived females showed lower Pi levels related to lower consumption of fizzy drinks, versus those drinking more than one fizzy drink a month (p=0.004, Table 3). There was no overt relationship between fizzy drink consumption and telomere length in this group (data not shown).

### Serum phosphate levels are associated with estimated glomerular filtration rate (eGFR) in pSoBid cohort

A relationship between Pi levels and eGFR (p=0.025, Table 2) was observed. This relationship showed a strong gender component, unaffected by deprivation status, with females having superior eGFR to males (95%CI −0.264 to −0.04; p=0.025).

**Figure 3:**
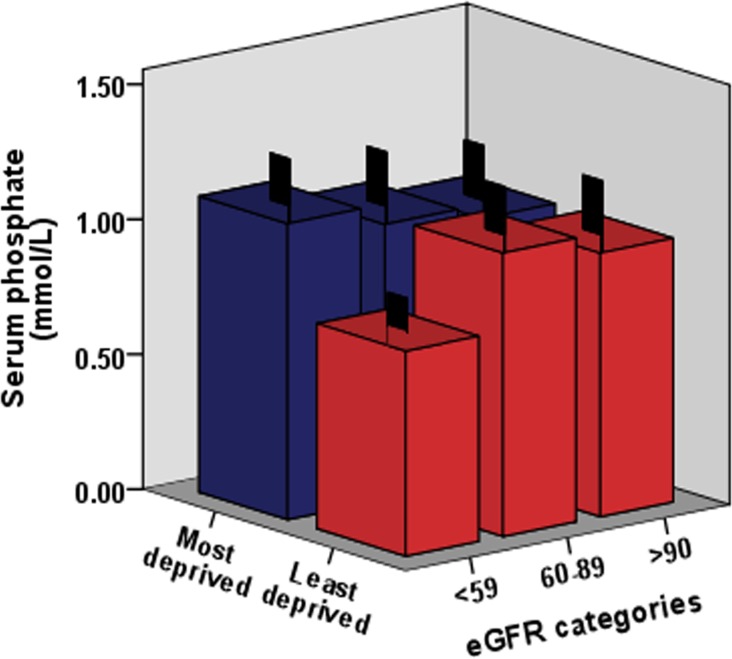
The relationship between Pi levels, eGFR (estimated glomerular filtration rate) and deprivation status in the pSoBid cohort eGFR categories were used to reflect overall mild to moderate kidney function decline and related to CKD status. The association of the serum phosphate levels and eGFR decline are shown in relation to deprivation status in males.

Analysis of Pi levels versus eGFR indicated a significant relationship, with a large number of subjects displaying eGFR below 90 ml/min per 1.73 m2, indicative of incipient kidney disease and early stage CKD. This observation is consistent with the relationships displayed with adiponectin and IL-6 levels. Figure 3 shows Pi levels and eGFR status (listed by the KDIGO system for CKD status classification) [24] in males in relation to deprivation status.

Over 60% of pSoBid cohort subjects analyzed presented with eGFR levels classified by KDIGO as having mild to moderate CKD, in association with their serum Pi levels (i.e. >89-60 mL/min per 1.73 m^2^= Stage 2, and >59-30 mL/min per 1.73 m^2^= Stage 3). When analysed by gender, the most deprived males again showed an increase in the prevalence of mild to moderate CKD relative to their least deprived counterparts (p=0.001).

Regular smokers also displayed higher serum Pi levels and showed an association with lower eGFR (95% CI −0.197 to −0.036, p=0.046).

## DISCUSSION

These data provide novel insight into a possible mechanism for the accelerated ageing and early onset disease observed amongst the most deprived in Glasgow.

Our data indicate that high Pi levels are associated with features of biological ageing, namely decreasing telomere length, increasing inflammatory status and genomic DNA hypomethylation [4, 5]. Similarly, Pi levels correlated with renal function, as determined by eGFR measurement, and parameters associated with insulin resistance, including adiponectin and cholesterol levels.

Notably, the correlation between Pi levels and accelerated biological ageing is prominent in males and most specifically in the most deprived males, where it is principally associated with increased red meat consumption. This effect appears to be independent of telomere attrition, as the association between Pi levels and telomere length is not strengthened as a result of adjustment for age and deprivation and gender. Deprived males also show other features consistent with accelerated biological ageing, including reduced genomic methylation content and elevated IL-6 levels.

No other foodstuff appears to adversely affect Pi levels in males. In females only fizzy drink consumption appeared to influence Pi levels. The association with fizzy drink consumption is striking; as those drinking less than one fizzy drink a month have significantly lower Pi levels than those consuming fizzy drinks more than once per month. This effect was strongest among the least deprived and no such association was found among the most deprived females or among males.

A gender difference in Pi levels was observed in this cohort. Females had higher Pi levels than males, a difference that increased with higher chronological age. This observation is non intuitive in the context of hyperphosphataemia being associated with decreased lifespan and health span in mammals [6], as human females in general, live longer than males, despite having higher Pi levels. One possible explanation for this is oestrogen status, and bone loss of Pi in post-menopausal females. Burnett-Bowie et al [25] have reported that the experimental suppression of testosterone levels in healthy volunteers was associated with increasing Pi concentration. Oestrogens are thought to facilitate the incorporation of Pi in remodeling bone; thus with advancing age Pi levels in women might increase.

The relationship between Pi levels and adiponectin is also striking. Adiponectin is typically a regulator of glucose metabolism and inflammatory signaling [26, 27] and may also influence bone turnover [28]. It is highest among the least deprived females, indicative of a better relative health status among this group and in keeping with a better health and longer lifespan. Notably, this group also possesses the greatest genomic methylation content and better eGFR than males, consistent with superior biological ageing relative to the other groups.

Our observations are consistent with a mechanism whereby elevated Pi is associated with increased CPP formation and cellular dysfunction and death. Moreover, among the most deprived elevated Pi is associated with high IL-6 levels, a measure of inflammatory status and increased comorbidity (Table 2) consistent with accelerated biological ageing.

That a Pi associated acceleration in biological ageing correlates with nutrition accords with the hypothesis that differences in biological ageing contribute to Glasgow's health disparity. Our finding that red meat consumption amongst the most deprived males correlated strongly with the advanced biological age in this group, relative to least deprived males and females of both socioeconomic groups, is novel. Notably, this association was not observed for other meat products (non-red meat including pates, sausages, haggis or ham). Red meat consumption has previously been related to increased cancer risk and overall mortality in a number of studies [29, 30]. Recent studies have reported that red meat consumption is associated with higher risk of cancer [31] and CVD [32]. In the present study, the observations indicate that elevated red meat consumption has adverse effects amongst a subject group that has a poor diet and sub optimal fruit and vegetable intake [4], where the effects of high Pi intake may be exacerbated. Notably, these effects are not apparent among less deprived males, or in females, especially in the context of a more balanced diet.

The prevalence of Pi containing additives is highest in low cost processed foods [33], most frequently consumed by the most deprived. Our study does not address this directly, but indicates that further study is merited for this aspect of diet. As Pi in beverages is readily absorbed these could also be postulated as having a significant impact on serum Pi levels. Fizzy drink consumption has recently been subject to much debate regarding the adverse impact on health from their elevated sugar content [34]. In this study, the effect of Pi levels from fizzy drink consumption was striking. This study indicates that the Pi content of these drinks actually had a limited impact on age related health. It should be noted, however that considerable amounts of Pi are also found in beer and wine [35] and this will need to be evaluated in more detail in pSoBid.

Strikingly, many of the subjects had eGFR values indicative of incipient or early onset CKD. Our observations are consistent with accelerated ageing [15, 16] being associated not only with premature vascular disease and inflammation but mild-moderate CKD [1–3]. These findings are directly relevant to the growing incidence of CKD globally [36, 37]. As the general Glaswegian population has a high prevalence of early stage CKD, especially so among the most deprived males. This indicates hyperphosphataemia associated with deprivation as a potential contributory factor to CKD. Indeed, it has been speculated that a high dietary Pi intake increases FGF-23 synthesis, which increases the Pi excretion per nephron. The Pi overload precipitates CaPi in renal tubular cells and leads to tubular injury and interstitial fibrosis, as extracellular Pi is toxic to the cell at high concentrations [38]. Indeed, data on 2269 healthy subjects from the Framingham Heart Study showed the odds ratio of developing CKD is much higher in healthy subjects with a serum Pi >4 mg/dL [39].

Our data suggest that Pi levels merit further study in larger and longitudinal cohorts, as well as in more controlled mechanistic studies. As high Pi intake has also been associated with increased mortality in an ostensibly healthy population [12]. Our findings may have important public health implications, both locally in Glasgow and in a wider public health context.

Weaknesses of the present study include its cross sectional nature and its limitation in size and limited data on type of red meat consumed (i.e. non processed beef, pork etc. versus processed meat). A number of potential confounders that were not taken into consideration also bear mentioning. While the genetics of the pSoBid cohort is relatively homogeneous, differences in epigenetics and circadian effects on ageing remain to be fully elucidated. These data are, however, biological plausible based on consistency with previous findings in this and other cohorts [17, 23, 38, 40]. In this respect, it is worth bearing in mind that analyses of large general population cohorts have inherent logistical issues with respect to the need for high throughput telomere analyses, hence the inclusion of techniques enabling individual telomere measurements, such as STELA, are not easily applied [41, 42], though the inclusion of an alternative technique in a sub set of samples, should enhance the robustness of any findings.

In summary, our data suggest that moderate hyper-phosphataemia promotes a gap between chronological and biological age. As high Pi intake is associated with increased mortality in an ostensibly healthy population [12] our findings have public health implications. In the light of both the recent non-domestic livestock red meat scandal and the WHO report on red meat consumption and cancer [33], it has not escaped our attention that red meat product quality and preservation may have an impact upon the diets of the most deprived and their associated health.

## MATERIALS AND METHODS

### pSoBid (Psychological, Social, and Biological Determinants of Ill Health) Cohort

Details of this cohort including biochemical parameters, physical examination and lifestyle questionnaires have been extensively described elsewhere [1–5]. Participants were ranked on the basis of multiple deprivation indicators using Scottish Index for Multiple Deprivation (SMID) and classified as least and most deprived in the NHS Greater Glasgow Health Board area. The sampling was designed to achieve an equal distribution across gender and age groups (35-44, 45-54 and 55-64) within the most (bottom 5% of SMID score) and least deprived areas (top 20% of SMID score). Information about food intake was collected from subjects as self-reported food frequency questionnaire incorporating, the information about 21 food categories and frequency of consumption (daily, weekly, monthly).

The serum samples were prepared from fasting blood and 604 remaining serum samples available from the 666 pSoBid participants were used in this study.

Ethical approval for the study was obtained from Glasgow Royal infirmary Research Ethic Committee and all participants gave written informed consent.

### Serum Phosphate analysis

Phosphate measurements were performed using an Instrumentation Laboratories ILAB 600 clinical chemistry analyser and the Inorganic Phosphorous Kit [Randox Laboratories (PH1016)] following the manufacturer's instructions. Assay calibration was performed using a specific calibrator supplied with the kit with an assigned value and validated with two levels of Quality Control material again supplied by Randox Assayed Multisera level 2 (HN1530_Level 3_(HE1532). Measurements were performed at 340nm.

### Relative Telomere Length

DNA was extracted from peripheral blood leukocytes using Maxwell®16 System (Maxwell® 16 Blood DNA Purification kit, Promega) and telomere lengths were determined by Q-PCR following the method of Cawthon. Telomere length analyses were performed in triplicate for each sample, using a single-copy gene amplicon primer set (acidic ribosomal phosphoprotein, 36B4) and a telomere-specific amplicon primer set. Quality control parameters, calculation of relative telomere length (T/S) and assay stability were reported previously [4].

### Global DNA methylation

Methylamp™Global DNA Methylation Quantification Ultra kit (Epigentek, USA) was used to estimate total DNA methylation as a percentage of total DNA present in the sample. The quality controls, methodology and assay QC was described previously [5].

### KDIGO classification

KDIGO 2012 Clinical Practice Guideline for the Evaluation and Management of Chronic Kidney Disease (CKD) [25] were used to estimate Glomerular Filtration Rate (eGFR) in the pSoBid cohort. The eGFR was classified as follows 1= Normal renal function, eGFR> 90 mL/min/1.73m^2^; CKD stage 2= eGFR 89-60 mL/min/1.73m^2^ and CKD stage 3 = eGFR 30-59 mL/min/1.73m^2^.

### Statistical analyses

Associations between biomarkers and Pi levels were analyzed using linear regression models. The model was adjusted using stratification variables by age, gender (Males and Females), deprivation (Most deprived and Least deprived) and annual income (<£25,000 and >£25,000). Prior to fitness to regression model, all the biomarkers were investigated for its approximation to normal distribution and natural log transformed when required. The association between biomarkers was quantified using Pearson's correlation coefficient (PCC) to determine its direction of relationship, and *p*-value for its test for significance. A critical threshold (p<0.05) was fixed which indicated a test to be statistically significant. Adjustment for the multiple comparisons was performed using Benjamini & Hochberg method. The confidence intervals were estimated by transforming PCC to z-score using Fishers transformation in order to estimate 95% confidence intervals (CI). The z-score were transformed back to corresponding *r* estimates that is associated to 95% CI. The frequency of food consumption was stratified by three different groups (G1.pD-G1.pW = greater than once per day – greater than once per week, L1.pW-L1.pM = less than once per week – less than once per month, G1.pM = greater than once per month. Kruskal-Wallis (KW) test was used to investigate the association between groups of food consumption frequency. Since KW test does not yield CIs, we bootstrapped KW test statistic by sampling data with replacement to report 95% CIs by calculating the percentiles (2.5%-97.5%) using the distribution of bootstrapped sampled test statistics.

## SUPPLEMENTARY TABLE


