# Supplementary material for: Accelerated ageing and renal dysfunction links lower socioeconomic status and dietary phosphate intake
Source: Aging (Albany NY). 2016 Apr 26;8(5):1135–48. doi: 10.18632/aging.100948 (PMC4931858; doi:10.18632/aging.100948)
Supplement: Supplementary file 1 [file aging-08-1135-s001.pdf]

SUPPLEMENTARY TABLE

Supplementary Table 1. Analysis of phosphate levels versus telomere length, DNA methylation content, and biochemical parameters with data adjusted for smoking status, alcohol consumption. Analysis adjusted for the multiple comparisons using the Benjamini-Hochberg method. eGFR - estimated glomerular filtration rate, BP-Blood pressure

Adjustment Covariates	Relative Telomere length	Global DNA methylation	Adiponectin	Cholesterol	Interleukin 6	eGFR (comb.ckdepi)	D -dimers	Vitamin D
**Regular smoking**	(n=360) r=-0.142 (-0.242 to -0.04) p=0.046*	(n=229) r=-0.234 (-0.353 to -0.107) p=0.016*	(n=586) r=0.114 (0.033 to 0.193) p=0.046*	(n=589) r=0.101 (0.02 to 0.18) p=0.057	(n=574) r=0.106 (0.024 to 0.186) p=0.052	(n=582) r=-0.117 (-0.197 to -0.036) p=0.046*	(n=583) r=0.121 (0.04 to 0.2) p=0.046*	(n=570) r=-0.08 (-0.161 to 0.002) p=0.164
**Alcohol**	(n=360) r=-0.484 (-0.559 to -0.401) p=0.42943	(n=229) r=-0.532 (-0.619 to -0.432) p=0.429	(n=586) r=0.426 (0.357 to 0.49) p=0.251	(n=589) r=0.424 (0.355 to 0.488) p=0.251	(n=574) r=0.431 (0.362 to 0.496) p=0.251	(n=582) r=-0.431 (-0.495 to -0.363) p=0.251	(n=583) r=0.424 (0.355 to 0.488) p=0.251	(n=570) r=-0.425 (-0.49 to -0.356) p=0.251
**High BP**	(n=357) r=-0.131 (-0.232 to -0.028) p=0.136	(n=227) r=-0.166 (-0.29 to -0.037) p=0.097	(n=583) r=0.104 (0.023 to 0.184) p=0.136	(n=586) r=0.089 (0.008 to 0.169) p=0.224	(n=571) r=0.123 (0.041 to 0.203) p=0.097	(n=579) r=-0.127 (-0.206 to -0.046) p=0.097	(n=580) r=0.126 (0.045 to 0.205) p=0.097	(n=567) r=-0.083 (-0.164 to -0.001) p=0.272
**Cancer**	(n=357) r=-0.193 (-0.291 to -0.091) p=0.004**	(n=227) r=-0.179 (-0.302 to -0.05) p=0.026*	(n=583) r=0.182 (0.102 to 0.259) p=0.0008***	(n=586) r=0.196 (0.117 to 0.272) p=0.0004***	(n=571) r=0.163 (0.082 to 0.242) p=0.002**	(n=579) r=-0.177 (-0.255 to -0.097) p=0.0008***	(n=580) r=0.172 (0.092 to 0.25) p=0.001**	(n=567) r=-0.158 (-0.237 to -0.077) p=0.003**
**Diabetes**	(n=357) r=-0.129 (-0.23 to -0.026) p=0.065	(n=227) r=-0.175 (-0.299 to -0.046) p=0.054	(n=583) r=0.125 (0.044 to 0.204) p=0.054	(n=586) r=0.112 (0.032 to 0.191) p=0.068	(n=571) r=0.127 (0.045 to 0.207) p=0.054	(n=579) r=-0.131 (-0.21 to -0.05) p=0.054	(n=580) r=0.136 (0.055 to 0.215) p=0.054	(n=567) r=-0.102 (-0.183 to -0.02) p=0.118
